# Dynamic Stability Analysis and Optimization of Multi-Vehicle Systems in Heterogeneous Connected Traffic

**DOI:** 10.3390/s25030727

**Published:** 2025-01-25

**Authors:** Tao Wang, Dayi Qu

**Affiliations:** 1 School of Mechanical and Automotive Engineering, Qingdao University of Technology, Qingdao 266520, China; wangtaoqd1987@163.com; 2 School of Artificial Intelligence and Big Data, Zibo Vocational Institute, Zibo 255300, China

**Keywords:** mixed-traffic flow, stability analysis, multi-vehicle interaction, car-following model

## Abstract

This study investigates the stability and performance of mixed-traffic flows consisting of human-driven vehicles (HDVs), connected autonomous vehicles (CAVs), autonomous vehicles (AVs), and connected human-driven vehicles (CHVs). Recognizing the complexity introduced by multi-vehicle interactions in such heterogeneous traffic, a refined CAV car-following model that integrates multi-vehicle state information, including headway, weighted velocity differences, weighted acceleration, and optimal velocity memory effects from both front and rear vehicles, is introduced. Through theoretical analysis of the model’s linear and nonlinear stability, the key parameters that enhance flow stability in mixed environments are determined. Numerical simulations across braking, start-up, and ring road scenarios validate the proposed model’s efficacy, demonstrating that it can effectively suppress traffic congestion and reduce oscillations, thereby improving traffic flow stability. This work offers valuable insights into the behavior of connected vehicles within mixed traffic and highlights the potential for CAV-based strategies to enhance both safety and efficiency in future transportation systems.

## 1. Introduction

The rapid evolution of transportation systems necessitates innovative approaches to enhance road safety and efficiency, particularly within the context of Vehicle-to-Everything (V2X) communication and dynamic vehicle interactions [1]. The integration of V2X technologies with autonomous driving systems has led to the emergence of connected autonomous vehicles (CAVs), which utilize Vehicle-to-Vehicle (V2V) communications to form interconnected networks. These networks enable the real-time exchange of critical traffic data—such as position, velocity, acceleration, and road conditions—thereby significantly improving early warning systems and reducing the likelihood of traffic accidents [2]. Moreover, V2X facilitates the exchange of real-time traffic information with cloud-based repositories, providing drivers with dynamic guidance and optimized route selection derived from robust traffic models. Empirical studies conducted under real-world traffic conditions have demonstrated that increasing the market penetration of CAVs substantially alleviates road congestion and elevates traffic safety standards [3]. However, before CAVs become ubiquitous, transportation networks will inevitably undergo a transitional phase characterized by a heterogeneous mix of vehicle types: human-driven vehicles (HDVs), connected human-driven vehicles (CHVs), autonomous vehicles (AVs), and connected autonomous vehicles (CAVs). In such a complex mixed-traffic environment, traditional traffic flow models and stability analysis methods become inadequate due to the diverse interaction dynamics among these vehicle categories [4]. Zong et al. [5] presented an adaptive car-following control framework designed to dynamically adjust between platoon and individual vehicle control based on surrounding traffic conditions. This framework aims to enhance the stability of CAV platoons, improve traffic efficiency, and reduce carbon emissions, while also considering the heterogeneous behavior of HDVs in mixed traffic. The study introduces a transposition prediction method to anticipate the reactions of rear vehicles, which helps CAVs form stable platoons and adapt to traffic disturbances.

Car-following models play a crucial role in characterizing the interactions and adaptive behaviors between vehicles, facilitating the prediction of traffic flow properties. These models often incorporate factors such as the distance between vehicles, relative velocity, and the diversity in the level of information availability among different types of vehicles [6]. Singh and Kumar [7] presented a new car-following model that incorporates a weighted average velocity field to account for multi-vehicle interactions in traffic flow. Stability analysis showed that the proposed model yielded a larger stability region compared to traditional models, and numerical simulations confirmed that incorporating a weighted average velocity reduced traffic congestion more effectively than a simple average approach. Chen et al. [8] introduced a Sigmoid-based car-following model designed to improve acceleration stability in mixed traffic, particularly addressing issues of traffic oscillation and following behavior in free-flow scenarios. Qin et al. [9] presented an energy-optimal car-following model for CAVs to improve traffic flow stability and reduce energy consumption. The model optimizes a key control parameter to stabilize mixed traffic consisting of CAVs and regular vehicles (RVs). Wu and Xiao [10] proposed an improved stochastic car-following model (SMLVM) that takes into account the complete state information of multiple preceding vehicles in a connected vehicle (CV) environment. The model integrates deterministic factors such as velocity, acceleration, headway, and optimal velocity memory while also incorporating stochastic influences through a Wiener process to simulate real-world uncertainties. Redhu et al. [11] proposed a novel car-following model that incorporates advanced driver reaction times within a V2X environment, particularly focusing on the effects of passing maneuvers and driver attention to neighboring vehicle velocities.

The continuous evolution of transportation systems, particularly with the integration of V2X communication technologies, necessitates innovative methodologies to enhance road safety and efficiency. In the context of modeling car-following behavior within V2X environments, a substantial body of research has been dedicated to modeling the car-following behavior of mixed traffic involving various vehicle types. For instance, Zhu et al. [12] investigated the impact of communication topologies on the dynamics of mixed-traffic flows, including HDVs and CAVs. They developed a generalized car-following model that integrates the effects of communication topologies, human reaction times, information delays, and optimal velocity changes with memory, and analyzed the linear stability of the system under different CT configurations. The results show that the multi-predecessor leader-following (MPLF) topology led to the best dynamic and steady performance, and that the concentration of CAVs at the front of the traffic flow significantly enhanced traffic stability. Wang et al. [13] introduced an autonomous driving car-following model based on safety distance principles, deriving fundamental diagrams of traffic flow under different penetration rates of Adaptive Cruise Control (ACC) and Cooperative Adaptive Cruise Control (CACC) vehicles. Xie et al. [14] developed a universal car-following model that simultaneously characterizes the behaviors of conventional vehicles and CVs, and established the linear stability conditions for mixed-traffic flows. Jiang et al. [15] investigated the effect of the maximum platoon size of CAVs on the safety of mixed-traffic flows, which include both HDVs and CAVs. The spatial distribution of different car-following models were analyzed using models such as the intelligent driver model (IDM), ACC, and CACC to simulate interactions between vehicles. Guo et al. [16] developed a freeway capacity model that incorporates both HDVs and CAVs, considering the behavioral traits of HDVs following CAV platoons. The simulation results indicate that driver behavior significantly influences capacity, with larger CAV platoons leading to more conservative following by HDVs. Zhu et al. [17] proposed a generalized car-following model to capture the dynamics of mixed-traffic flows. The study integrated human reaction times, information delays, and vehicle status data, employing a theoretical perturbation analysis to determine stability conditions based on varying CAV penetration rates and spatial distributions. Dong et al. [18] analyzed the impact of beyond-line-of-sight connectivity on the capacity and stability of mixed-traffic flow, consisting of CAVs and HDVs. Using an analytical approach based on Markov chain modeling and extended linear stability analysis, they evaluated how coordinated driving enabled by beyond-line-of-sight communication can enhance traffic capacity and stability. Yao et al. [19] presented a capacity analysis model for mixed-traffic flows that considers the heterogeneity and maximum platoon size of CVs. The probability distribution model for platoon sizes was developed, and a fundamental diagram to characterize mixed-traffic flow dynamics was derived.

Current studies on mixed-traffic flow modeling typically adopt one of two approaches: either applying distinct models for different types of vehicles [20,21,22,23,24,25,26], or using a unified model that distinguishes them by assigning different parameters [27,28,29,30]. The former approach may suffer from limited generalizability and increased computational burden, while the latter often inadequately represents the enhanced information availability for CAVs compared to HDVs, emphasizing the effect of individual parameters rather than their interactive influence.

In summary, while research efforts have consistently focused on enhancing the stability and effectiveness of car-following models, several challenges and limitations persist. For CV car-following models, it is essential to comprehensively yet efficiently integrate multi-vehicle information without redundancy. In mixed-traffic environments, the interactions among different vehicle types are more complex, and issues such as packet loss and communication failures may arise, necessitating novel following strategies for CVs. Additionally, understanding the relationship between the penetration rate of CVs and both the driving efficiency of mixed traffic and the stability of traffic flow is urgently needed. Meanwhile, as vehicle networking and autonomous driving technologies continue to evolve, roadways are expected to feature a combination of HDVs, CHVs, AVs, and CAVs. However, relatively few studies have explored the stability and simulation validation of complex mixed-traffic flows that include HDVs, CHVs, AVs, and CAVs.

To address these challenges, this study introduces a comprehensive modeling framework for mixed-traffic flows that includes HDVs, CAVs, AVs, and CHVs. The proposed approach integrates multi-vehicle state information—including headway, velocity difference, acceleration, and memory effects—into a unified model. This framework not only captures the interactive dynamics between different vehicle types but also explicitly incorporates the enhanced information acquisition capabilities of CAVs through V2V communication.

The proposed model overcomes the limited generalizability of distinct models by establishing a unified framework that accounts for the diverse interactions among vehicle types. By leveraging multi-vehicle state information, the model reduces computational redundancy while maintaining scalability across different traffic scenarios. Furthermore, the inclusion of memory effects and the interaction between front and rear vehicle parameters enhances the representation of vehicle behavior, providing a more nuanced depiction of mixed-traffic dynamics. This approach also addresses the limitations of existing unified models by distinguishing vehicle types not only through parameter variations but also through their interaction and contribution to overall traffic stability.

In summary, this study lays a foundation for improving the stability and efficiency of mixed-traffic flows by addressing the limitations of current modeling approaches. Through both linear and nonlinear stability analyses and extensive simulation experiments, this research demonstrates the potential of the proposed model to mitigate velocity oscillations, enhance flow stability, and adapt to the evolving demands of mixed-traffic environments.

## 2. Analysis of Car-Following Characteristics in Mixed-Traffic Flow

**Figure 1:**
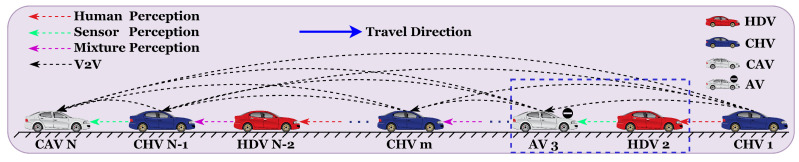
Mixed -traffic flow scenario.

In mixed-traffic environments consisting of HDVs, CAVs, AVs, and CHVs, the primary differences between these vehicle types lie in their control mechanisms and the way they acquire information. HDV drivers make decisions based on the operational states of adjacent vehicles and the surrounding environment but are generally insensitive to the acceleration changes of nearby vehicles. Consequently, modeling HDV car-following behavior typically considers only the immediate headway and relative velocity with the vehicle directly ahead or behind. Conversely, CAVs, AVs, and CHVs have access to more comprehensive information through onboard sensors, roadside units, and communication technology. CAVs, equipped with autonomous driving systems, execute driving tasks by obtaining multi-vehicle information such as velocity, headway, and acceleration from both front and rear vehicles through V2V communication technologies. AVs are treated as degraded versions of CAVs, controlled by their autonomous systems but experiencing degradation in one of three forms: front, rear, or both. In degraded segments, AVs use onboard sensors to collect information from multiple vehicles, while non-degraded parts continue to utilize V2V communications. CHVs are operated by human drivers but are augmented with V2V communication capabilities, allowing them to receive multi-vehicle information from surrounding traffic. The vehicle’s warning system provides the driver with pertinent driving recommendations based on these data. The typical mixed-traffic flow consisting of HDVs, CAVs, AVs, and CHVs is illustrated in Figure 1. In Figure 1, mixture perception refers to the way in which CHVs operate by integrating information from multiple sources. In addition to gathering data on surrounding vehicles via V2V communication, CHV drivers also rely on their own sensory perception to navigate, making decisions based on observed vehicular behavior and the information provided by onboard sensors.

### 2.1. Car-Following Model for HDVs

**Figure 2:**
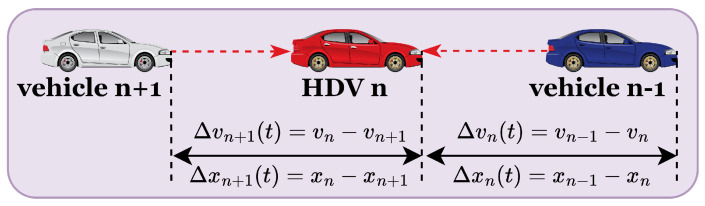
Sketch of HDVs.

In the context of HDVs, vehicle behavior is primarily determined by the perception and decisions of the driver, who relies on their sensory input to gather information about the status of the front and rear vehicle. As illustrated in Figure 2, HDV drivers adjust their driving behavior based on this information, without assistance from advanced communication technologies such as V2V.

The backward-looking effect, incorporated into the FVD model [31], expands traditional car-following paradigms by accounting for the behavior of vehicles behind the subject vehicle. This mechanism introduces a bidirectional influence, where the interactions between preceding and following vehicles contribute to a more comprehensive representation of traffic dynamics [20]. Specifically, this effect highlights how rear-vehicle behaviors impact the headway adjustments and stability characteristics of the leading vehicle.

Simultaneously, the memory effect, as discussed in prior studies [32], encapsulates the influence of historical driving information on current acceleration and deceleration decisions. It models drivers’ anticipatory adjustments based on previously experienced traffic conditions, allowing for enhanced stabilization of traffic flow by mitigating abrupt transitions caused by short-term fluctuations. This effect is particularly critical in representing the cognitive processes of human drivers, where memory of prior conditions aids in smoother velocity adjustments and improved overall traffic stability.

Incorporating both the backward-looking effect and the memory effect into the car-following model results in a more realistic and adaptive representation of HDV behavior:

(1) $$\begin{array}{cc} \frac{dv_{n}^{HDV}\left(t\right)}{dt} & =\alpha_{HDV}\left\{\epsilon V_{F}\left[\Delta x_{n}\left(t\right)\right]+\left(1-\epsilon \right)V_{B}\left[\Delta x_{n+1}\left(t\right)\right]-v_{n}\left(t\right)\right\}+\lambda_{HDV}\Delta v_{n}\left(t\right)\\ & +\gamma_{HDV}\left\{\epsilon V_{F}\left[\Delta x_{n}\left(t\right)\right]-\epsilon V_{F}\left[\Delta x_{n}\left(t-\tau \right)\right]+\left(1-\epsilon \right)V_{B}\left[\Delta x_{n+1}\left(t\right)\right]\right.\\ & \left.-\left(1-\epsilon \right)V_{B}\left[\Delta x_{n+1}\left(t-\tau \right)\right]\right\} \end{array}$$

where $\alpha_{HDV}$ represents the sensitivity coefficient to the optimal vehicle velocity; $\lambda_{HDV}$ denotes the sensitivity coefficient to the velocity difference between the vehicle *n* and its preceding vehicle n−1; $\Delta x_{n}\left(t\right)$ represents the headway distance between vehicle *n* and vehicle n−1 at time *t*; $v_{n}\left(t\right)$ denotes the velocity of vehicle *n* at time *t*; ϵ represents the degree of influence exerted by the preceding vehicle on the target vehicle, indicating how strongly the target vehicle’s behavior is affected by the vehicle ahead; τ represents the sampling interval; $\gamma_{HDV}$ represents the sensitivity coefficient for memory of driving tendencies, which quantifies how a vehicle’s past driving behavior influences its current decisions. $V_{F}\left(\cdot \right)$ and $V_{B}\left(\cdot \right)$ denote the optimal velocity functions relative to the front and rear vehicles, respectively. The specific expressions for these optimal velocity functions are as follows:

(2) $$\begin{array}{c} V_{F}\left[\Delta x_{n}\left(t\right)\right]=\alpha_{1}\left\{tanh\left[\Delta x_{n}\left(t\right)-h_{s}\right]+tanh\left(h_{s}\right)\right\} \end{array}$$

(3) $$\begin{array}{c} V_{B}\left[\Delta x_{n+1}\left(t\right)\right]=-\alpha_{2}\left\{tanh\left[\Delta x_{n+1}\left(t\right)-h_{s}\right]+tanh\left(h_{s}\right)\right\} \end{array}$$

where $\alpha_{1}$ and $\alpha_{2}$ represent velocity sensitivity coefficients; $h_{s}$ denotes the safe headway distance. To simplify the following analysis, based on [22], the following parameter values are adopted: $\alpha_{1}=1$, $\alpha_{2}=1$, and $h_{s}=4$.

To simplify the calculations, the nonlinear terms in the Taylor expansion of the variable $\Delta x_{n}\left(t-\tau \right)$ are disregarded. After this simplification, the expression is reduced to a more tractable form:

(4) $$\begin{array}{c} \Delta x_{n}\left(t-\tau \right)=\Delta x_{n}\left(t\right)-\tau \Delta v_{n}\left(t\right) \end{array}$$

Similarly, the following can be derived:

(5) $$\begin{array}{c} V_{F}\left[\Delta x_{n}\left(t-\tau \right)\right]=V_{F}\left[\Delta x_{n}\left(t\right)\right]-\tau \Delta v_{n}\left(t\right)V_{F}^{\prime }\left[\Delta x_{n}\left(t\right)\right] \end{array}$$

(6) $$\begin{array}{c} V_{B}\left[\Delta x_{n}\left(t-\tau \right)\right]=V_{B}\left[\Delta x_{n}\left(t\right)\right]-\tau \Delta v_{n}\left(t\right)V_{B}^{\prime }\left[\Delta x_{n}\left(t\right)\right] \end{array}$$

Finally, the following can be obtained:

(7) $$\begin{array}{cc} \frac{dv_{n}^{HDV}\left(t\right)}{dt} & =\alpha_{HDV}\left\{\epsilon V_{F}\left[\Delta x_{n}\left(t\right)\right]+\left(1-\epsilon \right)V_{B}\left[\Delta x_{n+1}\left(t\right)\right]-v_{n}\left(t\right)\right\}+\lambda_{HDV}\Delta v_{n}\left(t\right)\\ & +\gamma_{HDV}\left\{\epsilon \tau \Delta v_{n}\left(t\right)V_{F}^{\prime }\left[\Delta x_{n}\left(t\right)\right]+\left(1-\epsilon \right)\tau \Delta v_{n+1}\left(t\right)V_{B}^{\prime }\left[\Delta x_{n+1}\left(t\right)\right]\right\} \end{array}$$

### 2.2. Car-Following Model for CAVs

**Figure 3:**
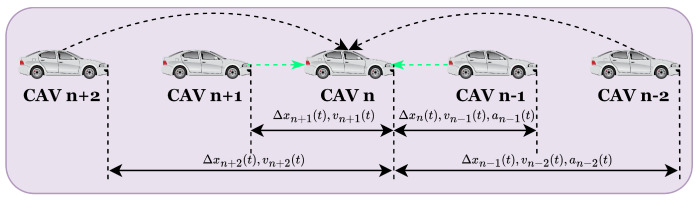
Sketch of CAVs.

Building upon the framework of V2V communication, CAVs can obtain comprehensive motion status information from all vehicles equipped with onboard V2V units, including both CAVs and CHVs. This information enables CAVs to automatically adjust their driving behavior based on real-time information from their surrounding environment, significantly enhancing safety and efficiency. Additionally, CAVs can also access the motion information of their nearest front and rear vehicle through onboard sensors, ensuring that the vehicle maintains a safe distance and adjusts its velocity accordingly (see Figure 3).

**Figure 4:**
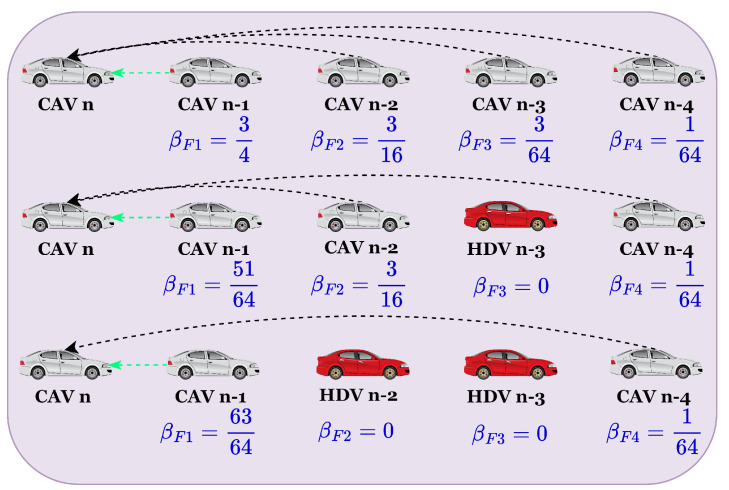
Illustration of weighting coefficients.

Considering the impact of information from multiple vehicles both ahead and behind, a refined car-following model (Weighted Multi-Vehicle Interaction Model with Memory, WMIMM) for CAVs has been developed as follows:

(8) $$\begin{array}{cc} \frac{dv_{n}^{CV}\left(t\right)}{dt} & =\alpha_{I}\left\{\epsilon V_{F}\left[\sum_{m=1}^{q}\beta_{Fm}\Delta x_{n-m+1}\left(t\right)\right]+\left(1-\epsilon \right)V_{B}\left[\sum_{m=1}^{p}\beta_{Bm}\Delta x_{n+m}\left(t\right)\right]-v_{n}\left(t\right)\right\}\\ & +\lambda_{I}\left[\sum_{m=1}^{q}\beta_{Fm}v_{n-m}\left(t\right)-v_{n}\left(t\right)\right]+\eta_{I}\left[\sum_{m=1}^{q}\frac{\beta_{Fm}}{m}a_{n-m}\left(t\right)\right]\\ & +\gamma_{I}\left\{\epsilon \tau \sum_{m=1}^{q}\sigma_{Fm}\Delta v_{n-m+1}\left(t\right)V_{F}^{\prime }\left[\Delta x_{n-m+1}\left(t\right)\right]\right.\\ & \left.+\left(1-\epsilon \right)\tau \sum_{m=1}^{p}\sigma_{Bm}\Delta v_{n+m}\left(t\right)V_{B}^{\prime }\left[\Delta x_{n+m}\left(t\right)\right]\right\} \end{array}$$

where $\Delta x_{n-m+1}$ represents the headway between vehicle n−m+1 and its preceding vehicle at time t; $v_{n-m}\left(t\right)$ and $a_{n-m}\left(t\right)$ represent the velocity and acceleration of vehicle n−m at time t; $\alpha_{I}$, $\lambda_{I}$, $\eta_{I}$, and $\gamma_{I}$ denote the sensitivity coefficient to optimal velocity, weighted velocity difference, weighted acceleration information, and memory of driving tendencies, respectively, where $I=\left\{CAV,AV\right\}$; $\sigma_{Fm}$ and $\sigma_{Bm}$ represent the sensitivity coefficient of *m*-th front and rear vehicles to the optimal velocity memory of driving tendencies; $\beta_{Fm}$ and $\beta_{Bm}$ represent the degree of influence the front and rear vehicles exerts on the target vehicle; *q* and *p* indicate the number of front and rear vehicles considered in the model. The weighting coefficients for the *m*-th vehicle are expressed as follows [17]:

(9) $$\begin{array}{c} \beta_{Fm},\sigma_{Fm}=\left\{\begin{array}{cc} \frac{C_{n,m}\left(q-1\right)}{q^{m}}, & m\in \left[2,q-1\right]\\ \frac{C_{n,q}}{q^{m-1}}, & m=q\\ 1-\frac{C_{n,q}}{q^{q-1}}-\sum_{i=2}^{q-1}\frac{C_{n,i}\left(q-1\right)}{q^{i-1}}, & m=1 \end{array}\right. \end{array}$$

(10) $$\begin{array}{c} \beta_{Bm},\sigma_{Bm}=\left\{\begin{array}{cc} \frac{C_{n,m}\left(p-1\right)}{p^{m}}, & m\in \left[2,p-1\right]\\ \frac{C_{n,p}}{p^{m-1}}, & m=p\\ 1-\frac{C_{n,p}}{p^{p-1}}-\sum_{i=2}^{p-1}\frac{C_{n,i}\left(p-1\right)}{p^{i-1}}, & m=1 \end{array}\right. \end{array}$$

where $C_{n,m}$ represents the communication connectivity parameter: $C_{n,m}=1$ indicates an active communication link between CAV *n* and vehicle *m*; otherwise, $C_{n,m}=0$, signifying no connection. To clarify the values of weighting coefficients, an example is shown in Figure 4. This parameter setup reflects that information from nearer vehicles carries a higher weight in data aggregation. If a preceding vehicle is out of communication range, its weight is reassigned to the nearest communicable downstream vehicle, ensuring uninterrupted information flow.

### 2.3. Car-Following Model for CHVs

For CHVs, driving decisions are influenced by a combination of factors. One key element is the driver’s own perception of the behavior of surrounding vehicles, which includes visual cues and personal judgment. In addition to this, CHVs benefit from V2V communication and sensor data, which provide real-time information about the position, velocity, and behavior of nearby vehicles. This dual source of information allows CHV drivers to make more informed and timely decisions during car-following scenarios. Consequently, the car-following model for CHVs is designed as follows:

(11) $$\begin{array}{c} \frac{dv_{n}^{CHV}\left(t\right)}{dt}=\theta \frac{dv_{n}^{CV}\left(t\right)}{dt}+\left(1-\theta \right)\frac{dv_{n}^{HDV}\left(t\right)}{dt} \end{array}$$

where $\theta \in \left[0,1\right]$ represents the compliance factor for V2V communication messages. When θ=0, it signifies that the target vehicle entirely disregards the information provided by V2V. For values of $\theta \in \left(0,1\right)$, a higher θ indicates an increasing degree of reliance on the V2V by the driver. Finally, when θ=1, it suggests that the vehicle exhibits full confidence in the V2V, relying completely on the data provided for decision-making.

### 2.4. Car-Following Models Considering Degradation Scenarios

In mixed-traffic flows, the spatial distribution of different types of vehicles can significantly influence the overall dynamics. This study assumes that CAVs and CHVs acquire information without considering communication delays. Additionally, it is presumed that CVs only experience degradation when the adjacent vehicle is an HDV, and in such cases, only the parameter $\alpha_{I}$ changes for degraded CAVs. Under these conditions, when the mixed-traffic flow reaches a stable state, all CAVs, regardless of whether they are degraded or not, are able to achieve the same optimal velocity. Through combined analysis, it is determined that six different car-following models exist within this complex mixed-traffic flow scenario:

1. CAVs with both the front and rear vehicles being CVs. The car-following models is given by Equation (8).
2. HDVs following other vehicles. The car-following models is given by Equation (7).
3. CAVs with both the front and rear vehicles being HDVs. The target CAV is positioned between two HDVs, and the CAV degrades to function as an AV. In this degraded state, the vehicle relies solely on onboard sensors to obtain information from multiple surrounding vehicles. Consequently, Equation (8) is simplified to the following expression to account for this degradation:
  (12) $$\begin{array}{cc} \frac{dv_{n}^{AV}\left(t\right)}{dt} & =\alpha_{AV}\left\{\epsilon V_{F}\left[\sum_{m=1}^{q}\beta_{Fm}\Delta x_{n-m+1}\left(t\right)\right]+\left(1-\epsilon \right)V_{B}\left[\sum_{m=1}^{p}\beta_{Bm}\Delta x_{n+m}\left(t\right)\right]-v_{n}\left(t\right)\right\}\\ & +\lambda_{CAV}\sum_{m=2}^{q}\beta_{Fm}v_{n-m}\left(t\right)+\lambda_{AV}\beta_{F1}\Delta v_{n}\left(t\right)+\eta_{CAV}\sum_{m=2}^{q}\frac{\beta_{Fm}}{m}a_{n-m}\left(t\right)\\ & +\eta_{AV}\beta_{F1}a_{n-1}\left(t\right)+\gamma_{I}\left\{\epsilon \tau \sum_{m=1}^{q}\sigma_{Fm}\Delta v_{n-m+1}\left(t\right)V_{F}^{\prime }\left[\Delta x_{n-m+1}\left(t\right)\right]\right.\\ & \left.+\left(1-\epsilon \right)\tau \sum_{m=1}^{p}\sigma_{Bm}\Delta v_{n+m}\left(t\right)V_{B}^{\prime }\left[\Delta x_{n+m}\left(t\right)\right]\right\} \end{array}$$
4. CAVs with only the preceding vehicle as an HDV. The target CAV has an HDV immediately ahead and a non-HDV vehicle behind, the CAV degrades to function as an AV relative to the vehicle in front. In this degraded state, it relies on onboard sensors to gather information from multiple preceding vehicles, while utilizing V2V communication to acquire data from multiple following vehicles. As a result, Equation (8) simplifies to the following form to reflect this degradation:
  (13) $$\begin{array}{cc} \frac{dv_{n}^{AVF}\left(t\right)}{dt} & =\alpha_{AV}\epsilon V_{F}\left[\sum_{m=1}^{q}\beta_{Fm}\Delta x_{n-m+1}\left(t\right)\right]+\alpha_{CAV}\left(1-\epsilon \right)V_{B}\left[\sum_{m=1}^{p}\beta_{Bm}\Delta x_{n+m}\left(t\right)\right]\\ & -\left(\alpha_{AV}\frac{\epsilon }{2\epsilon -1}+\alpha_{CAV}\frac{\epsilon -1}{2\epsilon -1}\right)v_{n}\left(t\right)+\lambda_{CAV}\sum_{m=2}^{q}\beta_{Fm}v_{n-m}\left(t\right)\\ & +\lambda_{AV}\beta_{F1}\Delta v_{n}\left(t\right)+\eta_{CAV}\sum_{m=2}^{q}\frac{\beta_{Fm}}{m}a_{n-m}\left(t\right)+\eta_{AV}\beta_{F1}\Delta a_{n-1}\left(t\right)\\ & +\gamma_{AV}\epsilon \tau \sum_{m=1}^{q}\sigma_{Fm}\Delta v_{n-m+1}\left(t\right)V_{F}^{\prime }\left[\Delta x_{n-m+1}\left(t\right)\right]\\ & +\gamma_{CAV}\left(1-\epsilon \right)\tau \sum_{m=1}^{p}\sigma_{Bm}\Delta v_{n+m}\left(t\right)V_{B}^{\prime }\left[\Delta x_{n+m}\left(t\right)\right] \end{array}$$
5. CAVs with only the following vehicle as an HDV. The CAV degrades to function as an AV relative to the vehicle behind. In this scenario, it relies solely on onboard sensors to gather information from multiple following vehicles, while utilizing V2V communication to obtain data from multiple preceding vehicles. As a result, Equation (8) simplifies to the following form to reflect this degradation:
  (14) $$\begin{array}{cc} \frac{dv_{n}^{AVB}\left(t\right)}{dt} & =\alpha_{CAV}\epsilon V_{F}\left[\sum_{m=1}^{q}\beta_{Fm}\Delta x_{n-m+1}\left(t\right)\right]+\alpha_{AV}\left(1-\epsilon \right)V_{B}\left[\sum_{m=1}^{p}\beta_{Bm}\Delta x_{n+m}\left(t\right)\right]\\ & -\left(\alpha_{CAV}\frac{\epsilon }{2\epsilon -1}+\alpha_{AV}\frac{\epsilon -1}{2\epsilon -1}\right)v_{n}\left(t\right)+\lambda_{CAV}\sum_{m=1}^{q}\beta_{Fm}v_{n-m}\left(t\right)\\ & +\eta_{CAV}\sum_{m=1}^{q}\frac{\beta_{Fm}}{m}a_{n-m}\left(t\right)+\gamma_{CAV}\epsilon \tau \sum_{m=1}^{q}\sigma_{Fm}\Delta v_{n-m+1}\left(t\right)V_{F}^{\prime }\left[\Delta x_{n-m+1}\left(t\right)\right]\\ & +\gamma_{AV}\left(1-\epsilon \right)\tau \sum_{m=1}^{p}\sigma_{Bm}\Delta v_{n+m}\left(t\right)V_{B}^{\prime }\left[\Delta x_{n+m}\left(t\right)\right] \end{array}$$
6. CHVs following other vehicles. The car-following models are given by Equation (11).

## 3. Stability Analysis

### 3.1. Linear Stability Analysis

This section delves into the stability analysis of the proposed WMIMM car-following model, with a focus on identifying the critical stability conditions related to its parameters. Additionally, the propagation characteristics of traffic flow under perturbations are examined from a microscopic perspective, as discussed in [33]. To evaluate the critical stability conditions of the WMIMM model, this study employs the method of small perturbations, as described in [34]. If the model is stable, any small disturbances introduced into the vehicle platoon will gradually dissipate, leading the traffic flow back to a stable equilibrium.

Assuming that the initial equilibrium headway between vehicles is uniformly set to $h_{e}$ and the velocity is $v_{e}$, with the position of the vehicle given by $X_{n}\left(t\right)=\left(n-1\right)h_{e}+v_{e}t$, a small disturbance in the form is introduced during the vehicle’s movement:

(15) $$\begin{array}{c} y_{n}\left(t\right)=e^{i\alpha_{k}n+zt}=x_{n}\left(t\right)-X_{n}\left(t\right) \end{array}$$

where *z* is the characteristic root, and $\alpha_{k}$ represents the *k*th Fourier expansion parameter.

To achieve the linearization of the equilibrium position, the first and second derivatives of Equation (15) are taken, yielding the corresponding vehicle-related variables as follows:

(16) $$\begin{array}{c} \left\{\begin{array}{c} x_{n}\left(t\right)=X_{n}\left(t\right)+y_{n}\left(t\right)\\ v_{n}\left(t\right)=v_{e}+{\dot{y}}_{n}\left(t\right)\\ a_{n}\left(t\right)={\ddot{y}}_{n}\left(t\right)\\ \Delta x_{n}\left(t\right)=h_{e}+\Delta y_{n}\left(t\right)\\ \Delta v_{n}\left(t\right)=\Delta {\dot{y}}_{n}\left(t\right) \end{array}\right. \end{array}$$

Based on (16) and by performing a first-order Taylor expansion of the proposed model around the equilibrium state of the traffic flow, the following expression is obtained:

(17) $$\begin{array}{cc} \frac{d^{2}y_{n}\left(t\right)}{dt^{2}} & =\alpha_{I}\left\{\epsilon V_{F}^{\prime }\left(h_{e}\right)\left(\sum_{m=1}^{q}\beta_{Fm}\Delta y_{n-m+1}\left(t\right)\right)+\left(1-\epsilon \right)V_{B}^{\prime }\left(h_{e}\right)\left(\sum_{m=1}^{p}\beta_{Bm}\Delta y_{n+m}\left(t\right)\right)-{\dot{y}}_{n}\left(t\right)\right\}\\ & +\lambda_{I}\left[\sum_{m=1}^{q}\beta_{Fm}{\dot{y}}_{n-m}\left(t\right)-{\dot{y}}_{n}\left(t\right)\right]+\eta_{I}\left[\sum_{m=1}^{q}\frac{\beta_{Fm}}{m}{\ddot{y}}_{n-m}\left(t\right)\right]\\ & +\gamma_{I}\left\{\epsilon \tau \sum_{m=1}^{q}\sigma_{Fm}\Delta {\dot{y}}_{n-m+1}\left(t\right)V_{F}^{\prime }\left(h_{e}\right)+\left(1-\epsilon \right)\tau \sum_{m=1}^{p}\sigma_{Bm}\Delta {\dot{y}}_{n+m}\left(t\right)V_{B}^{\prime }\left(h_{e}\right)\right\} \end{array}$$

From ${\dot{y}}_{n}\left(t\right)=ze^{i\alpha_{k}n+zt}$ and ${\ddot{y}}_{n}\left(t\right)=z^{2}e^{i\alpha_{k}n+zt}$, the following can be obtained:

(18) $$\begin{array}{cc} z^{2} & =\alpha_{I}\epsilon V_{F}^{\prime }\left(h_{e}\right)\sum_{m=1}^{q}\beta_{Fm}e^{-i\alpha_{k}\left(m-1\right)}\left(e^{-i\alpha_{k}}-1\right)+\alpha_{I}\left(1-\epsilon \right)V_{B}^{\prime }\left(h_{e}\right)\sum_{m=1}^{p}\beta_{Bm}e^{i\alpha_{k}\left(m-1\right)}\left(1-e^{i\alpha_{k}}\right)\\ & -\alpha_{I}z+\lambda_{I}z\left[\sum_{m=1}^{q}\beta_{Fm}e^{-i\alpha_{k}m}-1\right]+\eta_{I}z^{2}\left[\sum_{m=1}^{q}\frac{\beta_{Fm}}{m}e^{-i\alpha_{k}m}\right]\\ & +\gamma_{I}\epsilon \tau \sum_{m=1}^{q}\sigma_{Fm}ze^{-i\alpha_{k}\left(m-1\right)}\left(e^{-i\alpha_{k}}-1\right)V_{F}^{\prime }\left(h_{e}\right)\\ & +\gamma_{I}\left(1-\epsilon \right)\tau \sum_{m=1}^{p}\sigma_{Bm}ze^{i\alpha_{k}\left(m-1\right)}\left(1-e^{i\alpha_{k}}\right)V_{B}^{\prime }\left(h_{e}\right) \end{array}$$

Expanding $e^{i\alpha_{k}}$ using a second-order Taylor series yields $e^{i\alpha_{k}}=1+i\alpha_{k}+0.5{\left(i\alpha_{k}\right)}^{2}$. Substituting this expansion into Equation (18), we obtain

(19) $$\begin{array}{cc} z^{2} & =\alpha_{I}\epsilon V_{F}^{\prime }\left(h_{e}\right)\sum_{m=1}^{q}\beta_{Fm}\left(\left(2m-1\right)\frac{{\left(i\alpha_{k}\right)}^{2}}{2}-i\alpha_{k}\right)\\ & +\alpha_{I}\left(1-\epsilon \right)V_{B}^{\prime }\left(h_{e}\right)\sum_{m=1}^{p}\beta_{Bm}\left(\left(1-2m\right)\frac{{\left(i\alpha_{k}\right)}^{2}}{2}-i\alpha_{k}\right)-\alpha_{I}z\\ & +\lambda_{I}z\left[\sum_{m=1}^{q}\beta_{Fm}\left(\frac{{\left(i\alpha_{k}m\right)}^{2}}{2}-i\alpha_{k}m\right)\right]+\eta_{I}z^{2}\left[\sum_{m=1}^{q}\frac{\beta_{Fm}}{m}\left(\frac{{\left(i\alpha_{k}m\right)}^{2}}{2}-i\alpha_{k}m+1\right)\right]\\ & +\gamma_{I}\epsilon \tau \sum_{m=1}^{q}\sigma_{Fm}z\left(\left(2m-1\right)\frac{{\left(i\alpha_{k}\right)}^{2}}{2}-i\alpha_{k}\right)V_{F}^{\prime }\left(h_{e}\right)\\ & +\gamma_{I}\left(1-\epsilon \right)\tau \sum_{m=1}^{p}\sigma_{Bm}z\left(\left(1-2m\right)\frac{{\left(i\alpha_{k}\right)}^{2}}{2}-i\alpha_{k}\right)V_{B}^{\prime }\left(h_{e}\right) \end{array}$$

Expanding *z* as $z=z_{1}\left(i\alpha_{k}\right)+z_{2}{\left(i\alpha_{k}\right)}^{2}$ and substituting back into Equation (18), the first-order term $z_{1}$ and second-order term $z_{2}$ are obtained by equating the corresponding coefficients of $i\alpha_{k}$ and ${\left(i\alpha_{k}\right)}^{2}$:

(20) $$\begin{array}{c} z_{1}=-\epsilon V_{F}^{\prime }\left(h_{e}\right)-\left(1-\epsilon \right)V_{B}^{\prime }\left(h_{e}\right) \end{array}$$

(21) $$\begin{array}{cc} z_{2} & =\epsilon V_{F}^{\prime }\left(h_{e}\right)\left(\sum_{m=1}^{q}\beta_{Fm}\frac{2m-1}{2}\right)+\left(1-\epsilon \right)V_{B}^{\prime }\left(h_{e}\right)\left(\sum_{m=1}^{p}\beta_{Bm}\frac{1-2m}{2}\right)-\frac{\lambda_{I}\sum_{m=1}^{q}\beta_{Fm}m}{\alpha_{I}}z_{1}\\ & -\frac{\gamma_{I}\epsilon \tau V_{F}^{\prime }\left(h_{e}\right)}{\alpha_{I}}z_{1}-\frac{\gamma_{I}\left(1-\epsilon \right)\tau V_{B}^{\prime }\left(h_{e}\right)}{\alpha_{I}}z_{1}+\frac{\eta_{I}\sum_{m=1}^{q}\frac{\beta_{Fm}}{m}-1}{\alpha_{I}}z_{1}^{2} \end{array}$$

Notably, the expression of $z_{2}$ also contains the term $-\epsilon V_{F}^{\prime }\left(h_{e}\right)-\left(1-\epsilon \right)V_{B}^{\prime }\left(h_{e}\right)$. To simplify the final expression and reduce redundancy, $z_{1}$ is directly used to replace this term in $z_{2}$.

Based on the stability criterion [9], if $z_{2}<0$, the initially stable traffic flow becomes unstable due to the perturbation $y_{n}$. Conversely, when $z_{2}>0$, the disturbed traffic flow gradually returns to a stable state. Based on the expression for $z_{2}$ in Equation (21), the stability condition $z_{2}>0$ can be directly employed to analyze the sensitivity coefficient $\alpha_{I}$. Specifically, by isolating $\alpha_{I}$ from the inequality $z_{2}>0$, its range of values is derived as follows:

(22) $$\begin{array}{c} \alpha_{I}>\frac{\lambda_{I}\left(\sum_{m=1}^{q}\beta_{Fm}m\right)z_{1}+\left[1-\eta_{I}\left(\sum_{m=1}^{q}\frac{\beta_{Fm}}{m}\right)-\gamma_{I}\tau \right]z_{1}^{2}}{\epsilon V_{F}^{\prime }\left(h_{e}\right)\left(\sum_{m=1}^{q}\beta_{Fm}\frac{2m-1}{2}\right)+\left(1-\epsilon \right)V_{B}^{\prime }\left(h_{e}\right)\left(\sum_{m=1}^{p}\beta_{Bm}\frac{1-2m}{2}\right)} \end{array}$$

(23) $$\begin{array}{c} \alpha_{I}>\frac{\lambda_{I}z_{1}+\left[1-\gamma_{I}\tau \right]z_{1}^{2}}{0.5\epsilon V_{F}^{\prime }\left(h_{e}\right)-0.5\left(1-\epsilon \right)V_{B}^{\prime }\left(h_{e}\right)} \end{array}$$

To validate the stability performance of the proposed model, several representative models, such as the OVM [35], FVDM [31], OVCM [32], ITVDM [36], and BLMI [37], were selected for comparison. The stability criteria for these models are outlined as follows:

(24) $$\begin{array}{c} \alpha_{OVM}>2V_{F}^{\prime }\left(h_{e}\right) \end{array}$$

(25) $$\begin{array}{c} \alpha_{FVD}>2V_{F}^{\prime }\left(h_{e}\right)-2\lambda_{I} \end{array}$$

(26) $$\begin{array}{c} \alpha_{OVCM}>2\left(1-\gamma_{I}\tau \right)V_{F}^{\prime }\left(h_{e}\right)-2\lambda_{I} \end{array}$$

(27) $$\begin{array}{c} \alpha_{ITVDM}>\frac{2z_{1}^{2}+2z_{1}\lambda_{I}}{\epsilon V_{F}^{\prime }\left(h_{e}\right)-\left(1-\epsilon \right)V_{B}^{\prime }\left(h_{e}\right)} \end{array}$$

(28) $$\begin{array}{c} \alpha_{BLMI}>\frac{2z_{1}^{2}+z_{1}\lambda_{I}\left(q+1\right)}{\epsilon qV_{F}^{\prime }\left(h_{e}\right)-\left(1-\epsilon \right)V_{B}^{\prime }\left(h_{e}\right)} \end{array}$$

**Figure 5:**
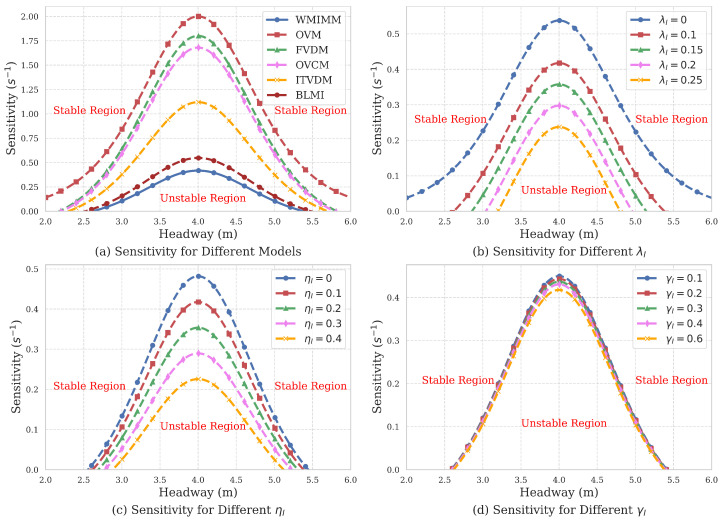
The stability curve plot of the model.

Based on Equation (22), a phase diagram can be constructed to illustrate the relationship between the headway $h_{e}$, and the sensitivity coefficients $\alpha_{I}$, $\lambda_{I}$, $\eta_{I}$, and $\gamma_{I}$. This phase diagram, as depicted in Figure 5, provides a comprehensive visual representation of how these factors interact and influence the system’s dynamic stability.

In Figure 5, the region above the curve represents the stable region, while the region below the curve indicates instability, where stop-and-go traffic phenomena occur. As shown in Figure 5a, the stability region of the WMIMM model is larger compared to other models, suggesting a higher likelihood of the traffic flow gradually returning to a stable state after disturbances. By examining the influence of sensitivity coefficients related to weighted velocity difference $\lambda_{I}$, weighted acceleration $\eta_{I}$, and memory effect on the model’s stability $\gamma_{I}$, as illustrated in Figure 5b–d, it can be observed that the WMIMM model enhances traffic flow stability through improved perception of weighted velocity difference with the preceding vehicles, weighted acceleration, and optimal velocity memory effect. This expanded sensing capability contributes significantly to the model’s robustness in maintaining smooth traffic flow, even under perturbations.

**Figure 6:**
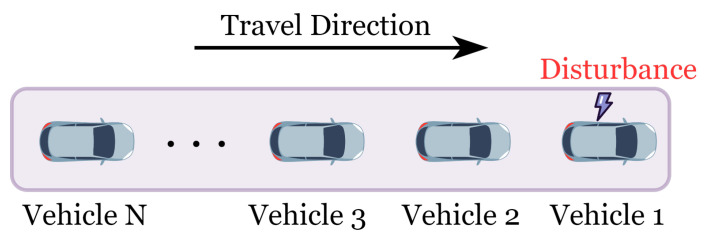
Scenario of comparative experiment.

In the vehicle operating scenario depicted in Figure 6, the initial headway is set to 4 m, with the lead vehicle subject to a velocity perturbation signal. The following model parameters were selected: α=0.41, ϵ=0.9, τ=0.2, λ=0.1, η=0.1, γ=0.3, and p=q=3.

Table 1 provides the distribution of vehicle velocity at the 100th sampling interval for various models, including maximum velocity $\left(V_{max}\right)$, average velocity $\left(V_{ave}\right)$, minimum velocity $\left(V_{min}\right)$, upward velocity fluctuation rate $\left(R_{up}\right)$, and downward velocity fluctuation rate $\left(R_{down}\right)$. The upward and downward velocity fluctuation rates are defined and calculated as follows:

(29) $$\left\{\begin{array}{c} R_{up}=\frac{1}{N}\sum_{i=1}^{N}\max\left(0,\frac{v_{i}-V_{ave}}{V_{ave}}\right)\times 100\%\\ R_{down}=\frac{1}{N}\sum_{i=1}^{N}\max\left(0,\frac{V_{ave}-v_{i}}{V_{ave}}\right)\times 100\% \end{array}\right.$$

From Table 1, it is evident that the WMIMM model exhibits an upward velocity fluctuation rate of 3.58% and a downward fluctuation rate of 5.27%, indicating relatively low fluctuation amplitudes in velocity control. Compared to other models, the WMIMM model achieves significant improvements in mitigating velocity variability. By integrating both back-looking effects and memory effects, along with multi-vehicle information from front and rear vehicles, the WMIMM model demonstrates superior stability and disturbance rejection performance over all other car-following models. These characteristics highlight the potential of the WMIMM model in enhancing vehicle and traffic flow control within mixed-traffic environments, underscoring its advantages in maintaining flow stability and resilience under mixed-traffic conditions.

### 3.2. Nonlinear Stability Analysis

The process of deriving linear stability conditions typically neglects second-order and higher-order nonlinear terms. According to Lyapunov’s first theorem, if the solution to the linearized system yields negative real eigenvalues, the system remains stable, and the ignored nonlinear terms do not lead to instability. However, if the linearized solution includes eigenvalues where some real parts are zero while the remaining real parts are negative, the omitted nonlinear terms can significantly influence the system’s overall stability and may lead to destabilizing effects [38]. Therefore, it is essential to conduct a nonlinear stability analysis near the critical stability conditions. The reductive perturbation method is employed to examine the nonlinear stability characteristics of the model. In this approach, gradual variables *X* and *T* are defined as follows [1,7]:

(30) $$\begin{array}{c} X=\mu \left(n+bt\right),T=\mu^{3}t \end{array}$$

where *b* represents the parameter to be determined, while μ denotes a small perturbation parameter, constrained within the range 0<μ≤1.

Equation (8) can be rewritten as follows:

(31) $$\begin{array}{cc} \frac{d^{2}\Delta x_{n}\left(t\right)}{dt^{2}} & =\alpha_{I}\left\{\epsilon V_{F}\left[\sum_{m=1}^{q}\beta_{Fm}\Delta x_{n-m}\left(t\right)\right]+\left(1-\epsilon \right)V_{B}\left[\sum_{m=1}^{p}\beta_{Bm}\Delta x_{n+m-1}\left(t\right)\right]\right.\\ & \left.-\epsilon V_{F}\left[\sum_{m=1}^{q}\beta_{Fm}\Delta x_{n-m+1}\left(t\right)\right]-\left(1-\epsilon \right)V_{B}\left[\sum_{m=1}^{p}\beta_{Bm}\Delta x_{n+m}\left(t\right)\right]-\Delta v_{n}\left(t\right)\right\}\\ & +\lambda_{I}\left[\sum_{m=1}^{q}\beta_{Fm}\Delta v_{n-m}\left(t\right)-\Delta v_{n}\left(t\right)\right]+\eta_{I}\left[\sum_{m=1}^{q}\frac{\beta_{Fm}}{m}\Delta a_{n-m}\left(t\right)\right]\\ & +\gamma_{I}\left\{\epsilon \tau \sum_{m=1}^{q}\sigma_{Fm}\Delta v_{n-m}\left(t\right)V_{F}^{\prime }\left[\Delta x_{n-m}\left(t\right)\right]\right.\\ & \left.+\left(1-\epsilon \right)\tau \sum_{m=1}^{p}\sigma_{Bm}\Delta v_{n+m-1}\left(t\right)V_{B}^{\prime }\left[\Delta x_{n+m-1}\left(t\right)\right]\right.\\ & \left.-\epsilon \tau \sum_{m=1}^{q}\sigma_{Fm}\Delta v_{n-m+1}\left(t\right)V_{F}^{\prime }\left[\Delta x_{n-m+1}\left(t\right)\right]\right.\\ & \left.-\left(1-\epsilon \right)\tau \sum_{m=1}^{p}\sigma_{Bm}\Delta v_{n+m}\left(t\right)V_{B}^{\prime }\left[\Delta x_{n+m}\left(t\right)\right]\right\} \end{array}$$

Let headway $\Delta x_{n}\left(t\right)=h_{s}+\mu R\left(X,T\right)$ and keep the carried out expansion to the fifth order in μ; then, the following equations can be obtained:

(32) $$\begin{array}{cc} & \mu^{2}\left[b+\epsilon \sum_{m=1}^{q}\beta_{Fm}V_{F}^{\prime }+\left(1-\epsilon \right)\sum_{m=1}^{p}\beta_{Bm}V_{B}^{\prime }\right]\partial_{X}R\\ & -\frac{\mu^{3}}{2}\left[\epsilon \sum_{m=1}^{q}\beta_{Fm}V_{F}^{\prime }\left(2m-1\right)-\left(1-\epsilon \right)\sum_{m=1}^{p}\beta_{Bm}V_{B}^{\prime }\left(2m-1\right)\right]\partial_{X}^{2}R\\ & +\frac{\mu^{3}}{\alpha_{I}}\left[b^{2}+b\lambda_{I}\sum_{m=1}^{q}\beta_{Fm}m-b^{2}\eta_{I}\sum_{m=1}^{q}\frac{\beta_{Fm}}{m}+\epsilon \tau \gamma_{I}bV_{F}^{\prime }+\left(1-\epsilon \right)\tau \gamma_{I}bV_{B}^{\prime }\right]\partial_{X}^{2}R\\ & +\frac{\mu^{4}}{6}\left[\epsilon \sum_{m=1}^{q}\beta_{Fm}V_{F}^{\prime }\left(3m^{2}-3m+1\right)+\left(1-\epsilon \right)\sum_{m=1}^{p}\beta_{Bm}V_{B}^{\prime }\left(3m^{2}-3m+1\right)\right]\partial_{X}^{3}R\\ & +\mu^{4}\left[\partial_{T}R+\frac{1}{6}\epsilon V_{F}^{\prime \prime \prime }\partial_{X}R^{3}+\frac{1}{6}\left(1-\epsilon \right)V_{B}^{\prime \prime \prime }\partial_{X}R^{3}-\frac{b\lambda_{I}}{2\alpha_{I}}\left(\sum_{m=1}^{q}\beta_{Fm}m^{2}\right)\partial_{X}^{3}R\right]\\ & +\frac{\mu^{4}}{\alpha_{I}}\left[b^{2}\eta_{I}-\frac{b\epsilon \tau \gamma_{I}}{2}\sum_{m=1}^{q}\sigma_{Fm}\left(2m-1\right)V_{F}^{\prime }+\frac{b\left(1-\epsilon \right)\tau \gamma_{I}}{2}\sum_{m=1}^{p}\sigma_{Bm}\left(2m-1\right)V_{B}^{\prime }\right]\partial_{X}^{3}R\\ & +\frac{\mu^{5}}{\alpha_{I}}\left[2b+\lambda_{I}\sum_{m=1}^{q}\beta_{Fm}m-2\eta_{I}b\sum_{m=1}^{q}\frac{\beta_{Fm}}{m}+\epsilon \tau \gamma_{I}V_{F}^{\prime }+\left(1-\epsilon \right)\tau \gamma_{I}V_{B}^{\prime }\right]\partial_{X}\partial_{T}R\\ & -\frac{\mu^{5}}{12}\left[\epsilon \sum_{m=1}^{q}\beta_{Fm}V_{F}^{\prime \prime \prime }\left(2m-1\right)-\left(1-\epsilon \right)\sum_{m=1}^{p}\beta_{Bm}V_{B}^{\prime \prime \prime }\left(2m-1\right)\right]\partial_{X}^{2}R^{3}\\ & +\frac{\mu^{5}}{24}\left[\frac{4}{\alpha_{I}}b\lambda_{I}\sum_{m=1}^{q}\beta_{Fm}m^{3}-12\frac{\eta_{I}}{\alpha_{I}}b^{2}\sum_{m=1}^{q}\beta_{Fm}m-\epsilon \sum_{m=1}^{q}\beta_{Fm}V_{F}^{\prime }\left(4m^{3}-6m^{2}+4m-1\right)\right.\\ & \left.+\left(1-\epsilon \right)\sum_{m=1}^{p}\beta_{Bm}V_{B}^{\prime }\left(4m^{3}-6m^{2}+4m-1\right)\right]\partial_{X}^{4}R+\frac{\mu^{5}}{6\alpha_{I}}\left[b\epsilon \tau \gamma_{I}\sum_{m=1}^{q}\sigma_{Fm}\left(3m^{2}-3m+1\right)V_{F}^{\prime }\right.\\ & \left.+b\left(1-\epsilon \right)\tau \gamma_{I}\sum_{m=1}^{p}\sigma_{Bm}\left(3m^{2}-3m+1\right)V_{B}^{\prime }\right]\partial_{X}^{4}R=0 \end{array}$$

Near the critical point $\left(h_{s},\alpha_{I}^{c}\right)$, let $\alpha_{I}^{c}=\alpha_{I}\left(1+\mu^{2}\right)$ and $b=-\epsilon \sum_{m=1}^{q}\beta_{Fm}V_{F}^{\prime }-\left(1-\epsilon \right)\sum_{m=1}^{p}\beta_{Bm}V_{B}^{\prime }$, and by applying these definitions, the second- and third-order terms involving μ are effectively canceled out in the equation, leading to the following simplified expression:

(33) $$\mu^{4}\left(\partial_{T}R-g_{1}\partial_{X}^{3}R+g_{2}\partial_{x}R^{3}\right)+\mu^{5}\left(g_{3}\partial_{X}^{2}R+g_{4}\partial_{X}^{4}R+g_{5}\partial_{X}^{2}R^{3}\right)=0$$

As a result, the coefficients associated with each term are specified as follows:

(34) $$\omega ≜\frac{1}{\alpha_{I}^{c}}\left[2b+\lambda_{I}\sum_{m=1}^{q}\beta_{Fm}m-2\eta_{I}b\sum_{m=1}^{q}\frac{\beta_{Fm}}{m}+\epsilon \tau \gamma_{I}V_{F}^{\prime }+\left(1-\epsilon \right)\tau \gamma_{I}V_{B}^{\prime }\right]$$

(35) $$\begin{array}{cc} g_{1} & =\frac{b\lambda_{I}}{2\alpha_{I}^{c}}\left(\sum_{m=1}^{q}\beta_{Fm}m^{2}\right)-\frac{1}{\alpha_{I}^{c}}\left[b^{2}\eta_{I}-\frac{b\epsilon \tau \gamma_{I}}{2}\sum_{m=1}^{q}\sigma_{Fm}\left(2m-1\right)V_{F}^{\prime }\right.\\ & \left.+\frac{b\left(1-\epsilon \right)\tau \gamma_{I}}{2}\sum_{m=1}^{p}\sigma_{Bm}\left(2m-1\right)V_{B}^{\prime }\right]-\frac{1}{6}\left[\epsilon \sum_{m=1}^{q}\beta_{Fm}V_{F}^{\prime }\left(3m^{2}-3m+1\right)\right.\\ & \left.+\left(1-\epsilon \right)\sum_{m=1}^{p}\beta_{Bm}V_{B}^{\prime }\left(3m^{2}-3m+1\right)\right] \end{array}$$

(36) $$g_{2}=\frac{1}{6}\epsilon V_{F}^{\prime \prime \prime }+\frac{1}{6}\left(1-\epsilon \right)V_{B}^{\prime \prime \prime }$$

(37) $$g_{3}=\frac{1}{\alpha_{I}^{c}}\left[b^{2}+b\lambda_{I}\sum_{m=1}^{q}\beta_{Fm}m-b^{2}\eta_{I}\sum_{m=1}^{q}\frac{\beta_{Fm}}{m}+\epsilon \tau \gamma_{I}bV_{F}^{\prime }+\left(1-\epsilon \right)\tau \gamma_{I}bV_{B}^{\prime }\right]$$

(38) $$\begin{array}{cc} g_{4} & =\omega g_{1}+\frac{1}{24}\left[\frac{4}{\alpha_{I}^{c}}b\lambda_{I}\sum_{m=1}^{q}\beta_{Fm}m^{3}-12\frac{\eta_{I}}{\alpha_{I}^{c}}b^{2}\sum_{m=1}^{q}\beta_{Fm}m-\epsilon \sum_{m=1}^{q}\beta_{Fm}V_{F}^{\prime }\left(4m^{3}-6m^{2}+4m-1\right)\right.\\ & \left.+\left(1-\epsilon \right)\sum_{m=1}^{p}\beta_{Bm}V_{B}^{\prime }\left(4m^{3}-6m^{2}+4m-1\right)\right]+\frac{1}{6\alpha_{I}^{c}}\left[b\epsilon \tau \gamma_{I}\sum_{m=1}^{q}\sigma_{Fm}\left(3m^{2}-3m+1\right)V_{F}^{\prime }\right.\\ & \left.+b\left(1-\epsilon \right)\tau \gamma_{I}\sum_{m=1}^{p}\sigma_{Bm}\left(3m^{2}-3m+1\right)V_{B}^{\prime }\right] \end{array}$$

(39) $$g_{5}=-\omega g_{2}-\frac{1}{12}\left[\epsilon \sum_{m=1}^{q}\beta_{Fm}V_{F}^{\prime \prime \prime }\left(2m-1\right)-\left(1-\epsilon \right)\sum_{m=1}^{p}\beta_{Bm}V_{B}^{\prime \prime \prime }\left(2m-1\right)\right]$$

To derive the standard modified Korteweg–de Vries (mKdV) equation that includes higher-order infinitesimal terms, the variable transformations $T=\frac{T^{\prime }}{g_{1}}$ and $R=R^{\prime }\sqrt{\frac{g_{1}}{g_{2}}}$ are selected. Substituting these expressions into Equation (33) allows us to obtain the standard mKdV equation augmented with higher-order correction terms:

(40) $$\partial_{T\prime }R^{\prime }-\partial_{X}^{3}R^{\prime }+\partial_{X}{R^{\prime }}^{3}+\mu \left(\frac{g_{3}\partial_{X}^{2}R^{\prime }}{g_{1}}+\frac{g_{4}\partial_{X}^{4}R^{\prime }}{g_{1}}+\frac{g_{5}\partial_{X}^{2}{R^{\prime }}^{3}}{g_{2}}\right)=0$$

By excluding the O(μ) terms from Equation (40), the standard form of the mKdV equation with its kink–antikink wave solution is expressed as follows:

(41) $$R_{0}^{\prime }\left(X,T^{\prime }\right)=\sqrt{c}\tanh\left[\sqrt{\frac{c}{2}}\left(X-cT^{\prime }\right)\right]$$

In order to determine the propagation velocity *c* of the kink wave in Equation (41), $R_{0}^{\prime }\left(X,T^{\prime }\right)$ must satisfy the following solvability condition:

(42) $$\left(R_{0}^{\prime },M\left[R_{0}^{\prime }\right]\right)=\int_{-\infty }^{+\infty }R_{0}^{\prime }\left(X,T^{\prime }\right)M\left[R_{0}^{\prime }\left(X,T^{\prime }\right)\right]dX=0$$

Then, integrating Equation (42) yields the propagation velocity of the kink wave as follows:

(43) $$c=\frac{5g_{2}g_{3}}{2g_{2}g_{4}-3g_{1}g_{5}}$$

Furthermore, by substituting the solution, the mKdV equation can be computed as follows:

(44) $$R\left(X,T\right)=\sqrt{\frac{g_{1}c}{g_{2}}\left(\frac{\alpha_{I}^{c}}{\alpha_{I}}-1\right)}\tanh\left\{\sqrt{\frac{c}{2}\left(\frac{\alpha_{I}^{c}}{\alpha_{I}}-1\right)}\left[n+\left(1-cg_{1}\left(\frac{\alpha_{I}^{c}}{\alpha_{I}}-1\right)t\right)\right]\right\}$$

where $A=\sqrt{\frac{g_{1}c}{g_{2}}\left(\frac{\alpha_{I}^{c}}{\alpha_{I}}-1\right)}$ is the amplitude of the wave.

The coexistence phase of traffic flow dynamics is characterized by the “kink—antikink” soliton solution, which encapsulates two primary states: free-flow and congested flow [39], mathematically expressed as follows:

(45) $$\Delta x_{n}\left(t\right)=h_{s}\pm A$$

**Figure 7:**
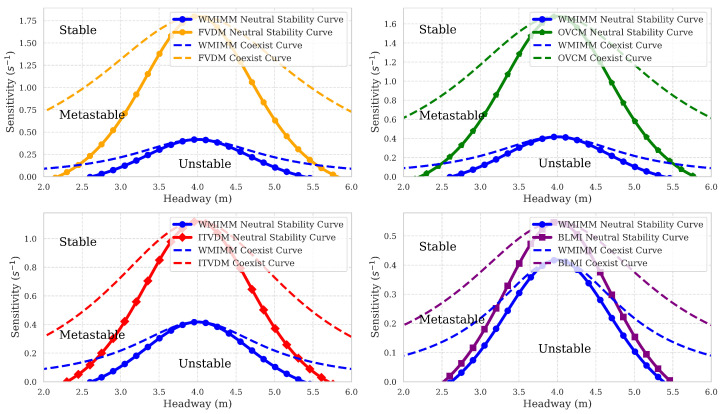
The comparison of coexist curve plot between different models.

The results from the nonlinear stability analysis illustrate the coexistence curves, as depicted for models like the WMIMM in Figure 7. The neutral stability curves of each model remain consistent with those shown in Figure 5. As observed in Figure 7, the neutral stability and coexistence curves divide the phase space into three distinct regions: the unstable region lies below the neutral stability curve, the metastable region exists between the neutral stability and coexistence curves, and the stable region is located above the coexistence curve. Within the metastable region, minor perturbations dissipate gradually, whereas larger disturbances may lead to traffic congestion. The figure reveals that the WMIMM model exhibits the largest stable region and the smallest metastable region. This demonstrates that incorporating multi-vehicle interaction significantly enhances traffic flow stability in this model.

## 4. Numerical Simulations

This study assumes that all vehicles on the road follow a car-following driving pattern, focusing solely on longitudinal behavior without considering lateral maneuvers, lane changes, pedestrian interactions, or other non-motorized traffic disturbances. The braking and start-up scenarios provide a straightforward reflection of car-following dynamics, encompassing a range of longitudinal behaviors observed in real traffic, such as queuing at red lights, accelerating on green signals, and emergency braking due to vehicle malfunctions. Additionally, a ring road setup, which lacks boundary conditions, better approximates continuous traffic flow and allows for a more direct evaluation of the proposed multi-vehicle state information cognition on traffic flow stability [40].

### 4.1. Sensitivity Analysis of Parameters of WMIMM Model in Ring Road Scenario

**Figure 8:**
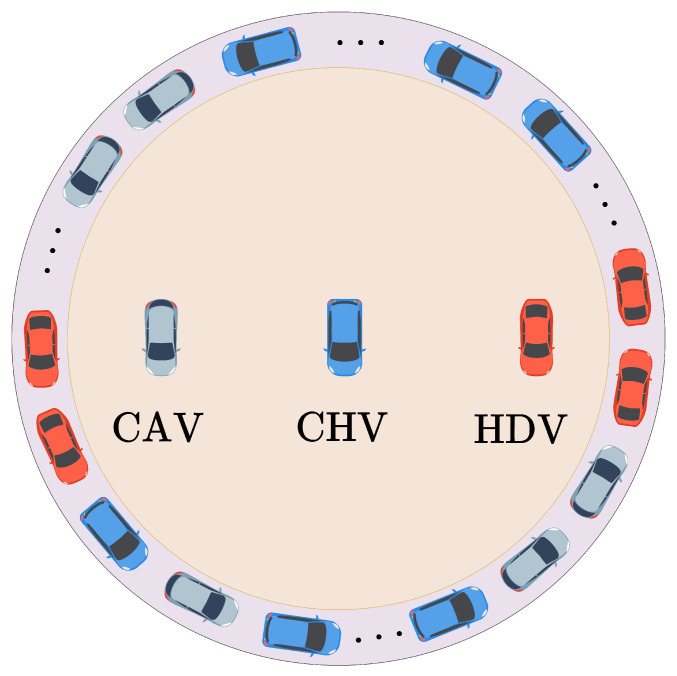
Ring road scenario.

In closed-loop configurations like a ring road, where vehicles form a uniformly distributed platoon, significant inter-vehicle constraints and prolonged disturbance propagation cycles are observed [30]. This setup demands highly responsive car-following models to effectively dampen disruptions across mixed-traffic flows of different types of vehicles. Within this closed-loop, single-lane configuration, each vehicle closely follows its predecessor, forming a continuous feedback loop as illustrated in Figure 8. Here, the state of the final vehicle directly influences the lead vehicle, creating a cyclic propagation of disturbances across all vehicles.

Considering a ring road with a total length of L=400 m, accommodating N=100 vehicles evenly spaced along its circumference. Each vehicle is assigned a unique identifier from 1 to 100, with the initial position of vehicle 1 injected into a disturbance which is set to $x_{1}\left(0\right)=1\;m$. The other position of the *n*-th vehicle is defined as $x_{n}\left(0\right)=\frac{(n-1)L}{N}m$, n∈[2,N]. Within this closed-loop structure, each vehicle has a designated following vehicle; for example, vehicle 1 follows directly behind vehicle 2, and vehicle *N* is positioned immediately in front of vehicle 1, thus maintaining a continuous flow around the ring. The arrangement of different types of vehicles is randomized throughout the platoon. The parameter settings for WMIMM model are outlined in Table 2:

#### 4.1.1. Velocity Fluctuations Influenced by ϵ

**Figure 9:**
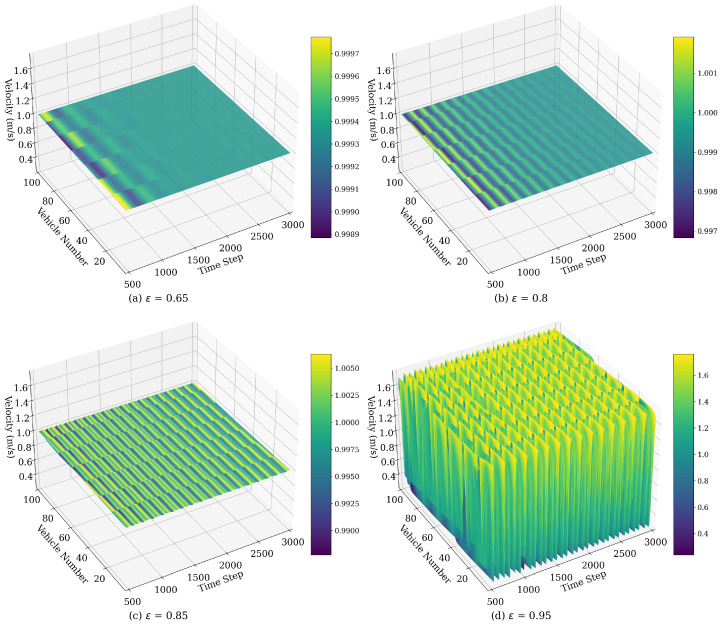
Space–time evolution of the velocity under different values of ϵ.

Figure 9 illustrates the spatiotemporal evolution of vehicle velocity under the WMIMM model after 500 time steps for varying values of ϵ. The following model parameters were selected: λ=0.1, η=0.1, and q=2. The traffic patterns depicted demonstrate the emergence and downstream propagation of stop-and-go traffic congestion following a minor perturbation introduced into the initially stable traffic flow.

In the cases where ϵ is high, such as ϵ=0.95, shown in Figure 9d, the focus on preceding vehicles leads to pronounced velocity oscillations throughout the traffic flow. Since each vehicle is predominantly influenced by those ahead, any fluctuation or disturbance is rapidly propagated forward, with limited dampening from rear vehicles. This lack of backward influence results in amplified responses to minor velocity changes, leading to a turbulent flow. In contrast, as ϵ decreases, the traffic flow dynamics shift towards a more balanced consideration of both preceding and rear vehicles. Higher backward sensitivity reduces velocity fluctuations by damping upstream disturbances, leading to a smoother, more uniform flow. At moderate to low values of ϵ, the traffic flow achieves stability, with minimal stop-and-go patterns as the backward effect effectively mitigates oscillations.

#### 4.1.2. Velocity Fluctuations Influenced by λ

**Figure 10:**
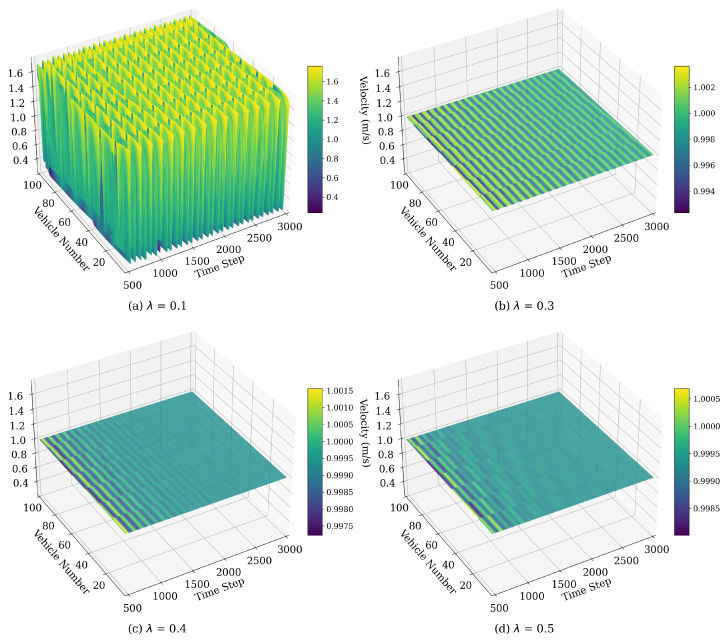
Space–time evolution of the velocity under different values of λ.

Figure 10 illustrates the spatiotemporal evolution of vehicle velocity based on the WMIMM model after 500 time steps under various values of parameter λ. The following model parameters were selected: ϵ=0.95, η=0.1, q=2, respectively. As seen in Figure 10a, there are pronounced oscillations in vehicle velocity with λ=0.1, resulting in a highly unstable flow characterized by strong stop-and-go waves. The instability arises because a low λ implies a reduced responsiveness to the weighted velocity difference between vehicles, causing disturbances to propagate downstream without sufficient correction. As λ increases from 0.3 to 0.5, traffic stability improves consistently. This trend demonstrates that higher λ values allow vehicles to respond more effectively to velocity differences, enabling faster correction of disturbances and promoting a uniform traffic flow. In summary, increasing λ progressively strengthens the traffic system’s ability to absorb and dissipate perturbations, ultimately resulting in a stable, homogeneous flow at sufficiently high values.

#### 4.1.3. Velocity Fluctuations Influenced by *q*

**Figure 11:**
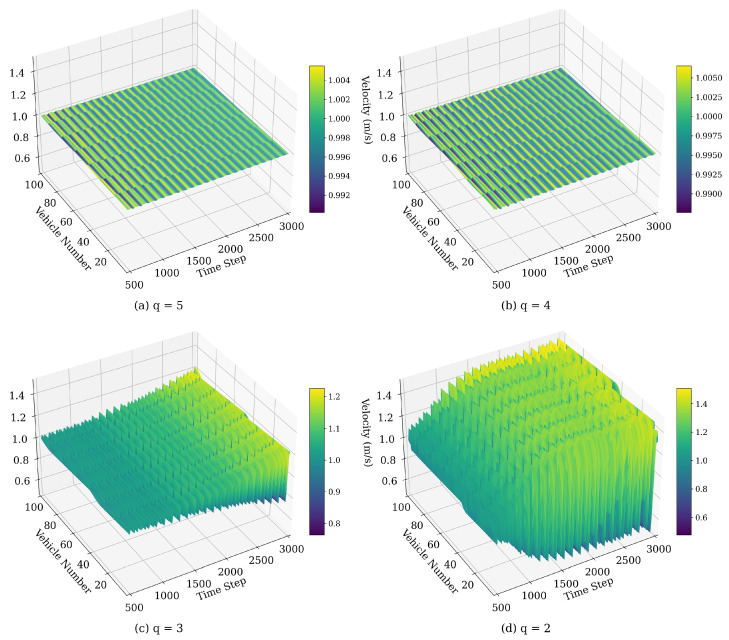
Space–time evolution of the velocity under different values of *q*.

The parameter *q*, representing the number of preceding vehicles considered in each vehicle’s velocity adjustment, significantly impacts the stability and fluctuation patterns of the traffic flow. The following model parameters were selected: ϵ=0.95, η=0.3, λ=0.2. As illustrated in Figure 11, increasing *q* generally enhances flow stability by incorporating more upstream information, which helps dampen stop-and-go oscillations. For example, at q=2, vehicle velocity fluctuations are pronounced, as each vehicle primarily reacts to a limited number of preceding vehicles. This limited information can lead to delayed responses to disturbances, allowing oscillations to propagate through the traffic flow. As *q* increases to 3 and beyond, the velocity fluctuations become progressively smoother. By considering more preceding vehicles, each vehicle better anticipates changes in traffic conditions, leading to a reduction in the amplitude of velocity oscillations.

#### 4.1.4. Velocity Fluctuations Influenced by η

**Figure 12:**
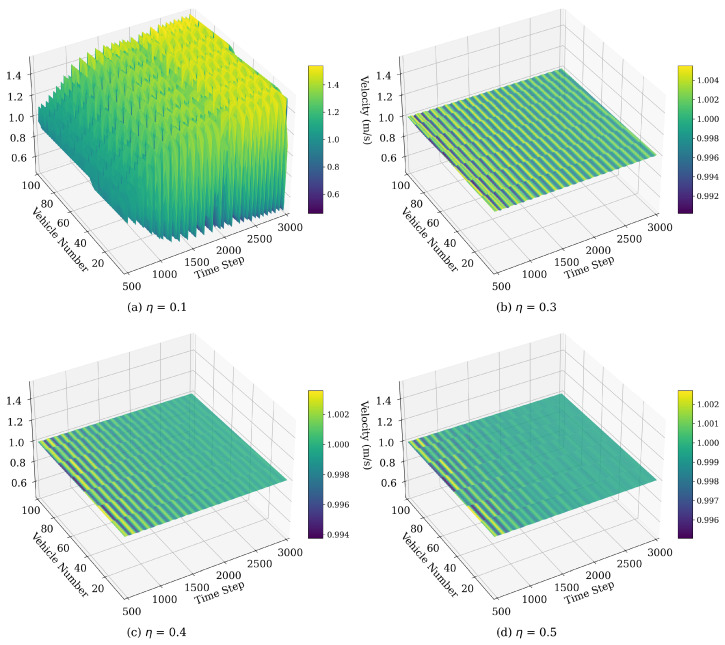
Space–time evolution of the velocity under different values of η.

The acceleration sensitivity coefficient η, which modulates the influence of multiple preceding vehicles’ accelerations, has a significant impact on traffic flow stability, as depicted in Figure 12. The following model parameters were selected: ϵ=0.95, q=3, λ=0.2. This parameter affects how sensitively a vehicle adjusts its velocity in response to the acceleration of nearby vehicles, thereby influencing the propagation of disturbances and overall flow dynamics. As η increases, vehicles adapt more quickly to changes in acceleration, effectively damping disturbances and reducing stop-and-go oscillations. Higher η values lead to smoother, more stable traffic flow, with minimal fluctuations, as vehicles better absorb and mitigate velocity variations. Thus, optimal traffic stability is achieved at moderate to high η values, which enhance responsiveness and minimize velocity disturbances.

#### 4.1.5. Velocity Fluctuations Influenced by the Ratio of CVs

**Figure 13:**
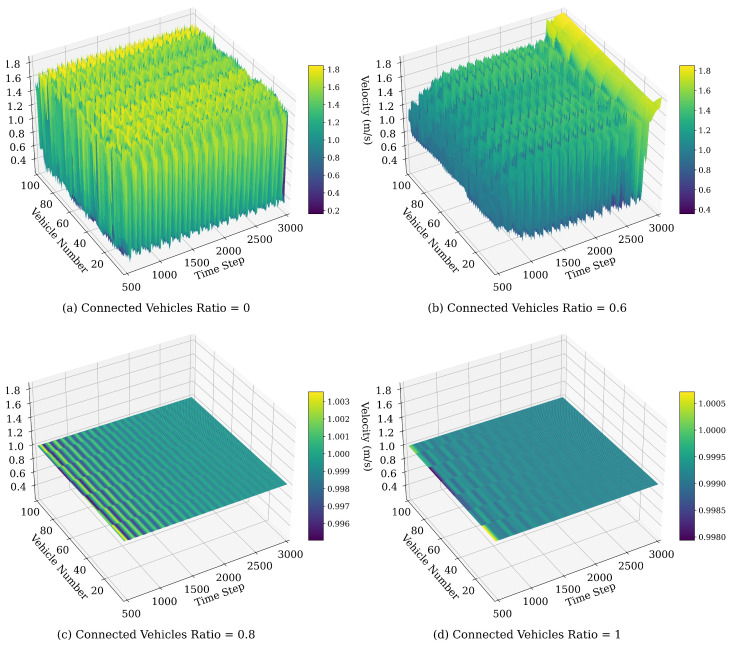
Space–time evolution of the velocity under different values of connected vehicles ratio.

Figure 13 illustrates the impact of varying CV penetration rates on the velocity fluctuation dynamics of vehicles in the ring road setting. Here, the term CV encompasses both CAVs and CHVs, with the remaining vehicles classified as HDVs. At lower connected vehicle penetration rates, such as 0 and 0.6, traffic flow exhibits significant velocity oscillations. This variability arises because the limited number of CVs, with their advanced communication and adaptive capabilities, is insufficient to counteract the less responsive behavior of HDVs. As the CV ratio increases to 0.8, velocity fluctuations diminish, as CVs’ communication and coordination capabilities help stabilize the flow by mitigating disruptions caused by HDVs. At a full CV ratio, velocity oscillations are eliminated, and the traffic flow approaches a steady state, with real-time communication and predictive control effectively suppressing disturbances. Thus, increasing CV penetration enhances traffic stability and efficiency, underscoring the value of CVs in achieving smooth and resilient traffic flow.

### 4.2. Braking and Start-Up Scenarios in Mixed Traffic

**Figure 14:**
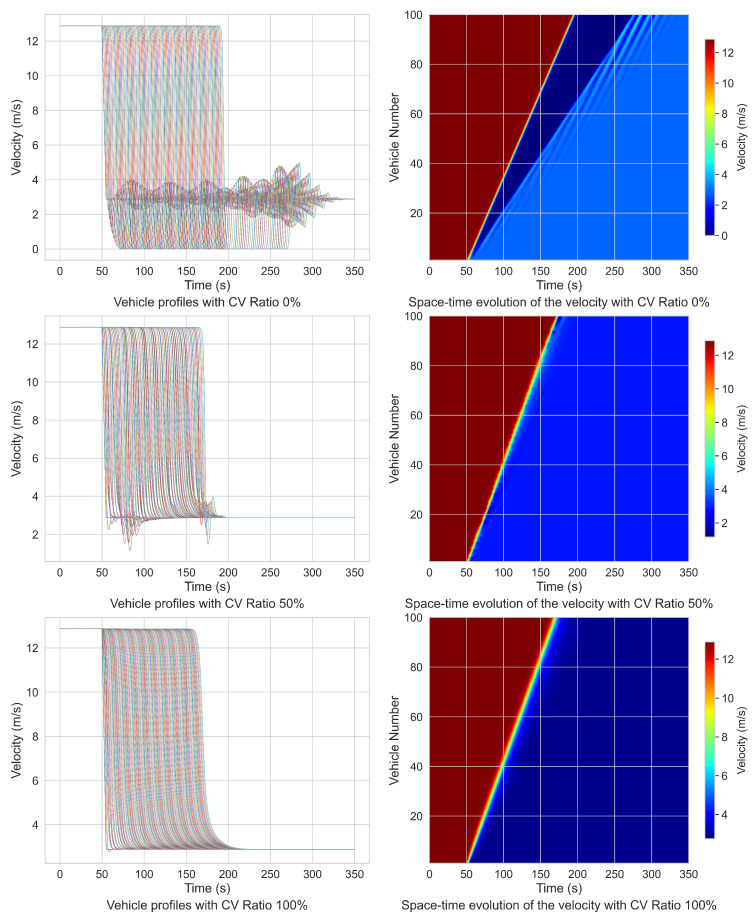
Velocity profiles in braking process with different connected vehicles ratio.

The simulation of the braking process for the mixed-traffic flow is designed as follows: assume an initial condition where 100 vehicles are traveling at a uniform velocity of $12.88\;m\cdot s^{-1}$. At time t=0, the position of each vehicle is defined by $x_{n}\left(0\right)=\left(n-1\right)\Delta x_{n}\left(t\right)$, where n=1,2,⋯,100, and the initial headway is set to $\Delta x_{n}\left(0\right)=24\;m$. At t=50 s, the lead vehicle initiates deceleration at a rate of $-2\;m\cdot s^{-2}$, and this deceleration is maintained for 5 seconds, after which the lead vehicle resumes driving at the perturbed velocity. Simulations are conducted with CV penetration rates of 0%, 50%, and 100%. Table 3 provides a comparative analysis of parameters for the braking process, including the calculated wave velocity, while Figure 14 illustrates the velocity profiles of individual vehicles, which represent the velocities within the platoon over time.

In this context, wave velocity refers to the propagation speed of perturbations (e.g., deceleration or acceleration signals) through the vehicle platoon. It is calculated by determining the time delay between the lead vehicle and the last vehicle responding to a disturbance. The wave velocity metric captures the efficiency with which information is transferred through the vehicle string and serves as a measure of traffic stability. Higher wave velocities indicate more rapid disturbance dissipation, reflecting improved traffic flow dynamics and stability.

As shown in Table 3 and Figure 14, the integration of CVs in a mixed-traffic environment effectively mitigates the propagation of velocity disturbances caused by braking events within the platoon. Specifically, at a 50% CV ratio, the fluctuation in acceleration was reduced by 37.59% compared to a fully HDV-based platoon, while the wave velocity of platoon increases by 19.23%. At a full CV ratio of 100%, the fluctuation in acceleration decreases by 63.15%, and the wave velocity increases by 25.98%. These results indicate that CVs have the capacity to regulate the overall platoon velocity, facilitating a smoother and faster braking process. In summary, the proposed car-following model, enables mixed traffic to complete braking processes more efficiently. By incorporating multi-vehicle state information, CVs can seamlessly assess the dynamic behavior of surrounding vehicles and perform braking control based on these data. Consequently, when CAVs operate within a mixed-traffic flow, the overall platoon deceleration duration is shortened, thereby reducing the total time required for the entire vehicle platoon to attain a new equilibrium state.

**Figure 15:**
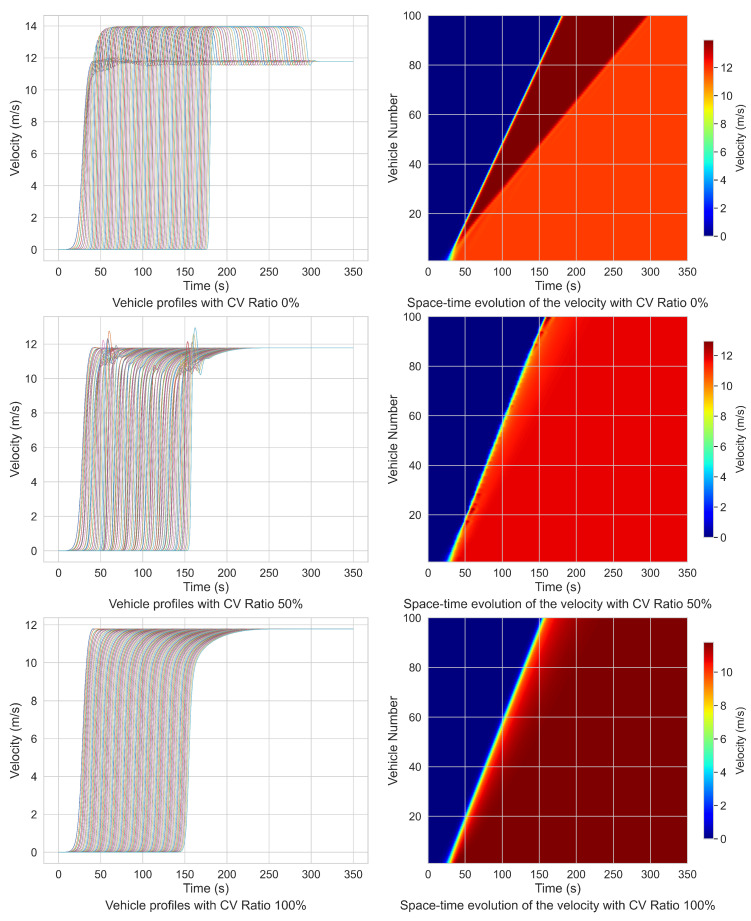
Velocity profiles in start-up process with different connected vehicles ratio.

Similarly, the start-up scenario is constructed as follows: the initial velocities of all 100 vehicles are set to zero, with their positions defined as the same in the braking scenario. At t=0, the initial headway is set to 6.3 m and the lead vehicle begins to accelerate with the profile based on [17], i.e., $\frac{12}{1+\exp(-0.35t+10)},t\in \left[0,40\;s\right]$, continuing this acceleration until reaching a constant cruising velocity of $12\;m\cdot s^{-1}$, after which it maintains this velocity. As the lead vehicle accelerates, the remaining vehicles in the platoon sequentially initiate their movement. All other parameters remain consistent with those in the braking process analysis, and simulations are conducted with CV penetration rates of 0%, 50%, and 100%. The resulting velocity profiles of the platoon are illustrated in Table 4 and Figure 15.

According to Table 4, when the CV penetration rate is set to 50%, the fluctuation in acceleration of the platoon is reduced by 26.53% compared to a fully HDV scenario, and the acceleration wave velocity rises by 16.37%. At a full CV penetration rate of 100%, the fluctuation in acceleration decreases by 57.14% relative to the all-HDV condition, and the wave propagation velocity of vehicle start-up improves by 22.5%. These findings indicate that CVs, within a mixed-traffic environment, are capable of adjusting the platoon’s overall velocity with reduced headways and propagating the wave more rapidly. This facilitates a smoother and faster platoon acceleration process, enabling vehicles to reach steady cruising velocities in a shorter time. Additionally, the integration of CVs into mixed-traffic flows contributes to an overall increase in acceleration rates, a reduction in acceleration fluctuation time, and a consequent decrease in the time required for the entire platoon to transition from standstill to uniform motion.

## 5. Conclusions

This study addresses the complexities of mixed-traffic flow composed of HDVs, CAVs, AVs, and CHVs by integrating the characteristics of CAVs and CHVs, as well as the influence of multi-vehicle information from both front and rear vehicles on various car-following modes. Specifically, for CVs, a CAV car-following model that incorporates multi-vehicle interaction information is proposed. This model utilizes inputs such as headway, weighted velocity difference, weighted acceleration, and memory effects from multiple front and rear vehicles. By fusing this interaction information, the proposed CAV car-following model effectively suppresses traffic congestion and enhances the stability of mixed-traffic flow in complex environments.

Through a theoretical analysis of the linear and nonlinear stability of the model, the stabilizing effects of incorporating multi-vehicle interaction information into traffic flow are determined. The effectiveness of the proposed car-following strategies and control methods is validated in scenarios including braking, start-up, and ring road conditions. By judiciously integrating information from multiple vehicles around CAVs, the model enables cooperative driving among vehicles in a mixed-traffic environment. This approach significantly mitigates the propagation of internal disturbances within the platoon, thereby enhancing the efficiency and stability of the mixed-traffic flow.

In future transportation systems, mixed-traffic environments where vehicles with varying levels of intelligence coexist are expected to become the norm. Given the current limitations on deploying real CAVs in mixed-traffic settings for large-scale, real-world testing, this model provides a feasible approach for simulating car-following behaviors within a mixed-traffic environment. Such simulations offer a theoretical foundation and model framework that can support future advancements in intelligent road traffic management and the deployment of digital transportation infrastructure. Future work will also benefit from the use of empirical data from real-world CAV experiments. Such data will enable a more accurate reconstruction of the model, enhancing its ability to reflect the operational dynamics of actual mixed-traffic environments.

## Figures and Tables

**Table 1:** Comparison of model stability parameters.

Model	$V_{\max}\;\left(m\cdot s^{-1}\right)$	$V_{\min}\;\left(m\cdot s^{-1}\right)$	$V_{\mathrm{ave}}\;\left(m\cdot s^{-1}\right)$	$R_{\mathrm{up}}\;\left(\%\right)$	$R_{\mathrm{down}}\;\left(\%\right)$
FVDM	1.123	0.931	0.995	9.21	11.03
OVCM	1.091	0.897	0.998	8.12	10.55
ITVDM	1.021	0.885	0.991	6.84	10.52
BLMI	0.863	0.732	0.811	5.12	9.68
WMIMM	0.816	0.761	0.792	3.58	5.27

**Table 2:** Parameter settings for WMIMM model simulation.

Parameter Names and Symbols	Values and Ranges
Safe Headway Distance $h_{s}$	4 m
Time Step Δt	0.2 s
Optimal Velocity Sensitivity Coefficient α	0.3
Optimal Velocity Weight of Multiple Vehicles ϵ	$\left[0.65,0.8,0.85,0.95\right]$
Velocity Difference Sensitivity Coefficient λ	$\left[0.1,0.3,0.4,0.5\right]$
Optimal Velocity Memory Sensitivity Coefficient γ	0.3
Number of Preceding Vehicles *q*	$\left[2,3,4,5\right]$
Number of Rear Vehicles *p*	2
Acceleration Sensitivity Coefficient of Multiple Vehicles η	$\left[0.1,0.3,0.4,0.5\right]$

**Table 3:** Parameter comparison of braking process under different CV ratios.

CV Ratio (%)	Average Acceleration $\left(m\cdot s^{-2}\right)$	Wave Velocity $\left(m\cdot s^{-1}\right)$
0	0.133	16.90
50	0.083	20.15
100	0.049	21.29

**Table 4:** Parameter comparison of start-up process under different CV ratios.

CV Ratio (%)	Average Acceleration $\left(m\cdot s^{-2}\right)$	Wave Velocity $\left(m\cdot s^{-1}\right)$
0	0.049	3.91
50	0.036	4.55
100	0.021	4.79

## Data Availability

Data are contained within the article.
